# ﻿The addition of six novel species and a new record of *Amphisphaeria* from northern Thailand

**DOI:** 10.3897/mycokeys.125.163523

**Published:** 2025-11-14

**Authors:** Zaw Lin Tun, Digvijayini Bundhun, Chitrabhanu S. Bhunjun, Sajeewa S. N. Maharachchikumbura, Vinodhini Thiyagaraja, Fatimah Al-Otibi, Kevin D. Hyde

**Affiliations:** 1 CAS Key Laboratory for Plant Diversity and Biogeography of East Asia, Kunming Institute of Botany, Chinese Academy of Science, Kunming, Yunnan 650201, China Mae Fah Luang University Chiang Rai Thailand; 2 School of Science, Mae Fah Luang University, Chiang Rai 57100, Thailand Chinese Academy of Science Kunming China; 3 Mushroom Research Foundation, 128 M.3 Ban Pa Deng T. Pa Pae, A. Mae Taeng, Chiang Mai 50150, Thailand Mushroom Research Foundation Chiang Mai Thailand; 4 Center of Excellence in Fungal Research, Mae Fah Luang University, Chiang Rai 57100, Thailand University of Electronic Science and Technology of China Chengdu China; 5 School of Life Science and Technology, University of Electronic Science and Technology of China, Chengdu 611731, China Uva Wellassa University Badulla Sri Lanka; 6 Department of Biosystems Technological Studies, Faculty of Technological Studies, Uva Wellassa University, Badulla, Sri Lanka Chinese Academy of Sciences Kunming China; 7 Department of Botany and Microbiology, College of Science, King Saud University, P.O. Box 22452, Riyadh 11495, Saudi Arabia King Saud University Riyadh Saudi Arabia; 8 Department of Plant Pathology, College of Agriculture, Guizhou University, Guiyang Guizhou 550025, China Guizhou University Guiyang China

**Keywords:** 6 novel species, inconspicuous taxa, Sordariomycetes, woody twigs

## Abstract

This study introduces six new *Amphisphaeria* species, discovered on dead twigs belonging to Calophyllaceae, Fabaceae, Sapotaceae, and Theaceae in northern Thailand, based on a morpho-phylogenetic approach. Our newly introduced *Amphisphaeria* species share morphological traits with other members of the genus, featuring ascomata with a two-layered peridium and unitunicate asci with either J+ or J- apical ring. The newly identified species *Amphisphaeria mesuae*, *A. mimusopis*, *A. paraserianthis*, *A. pseudomicheliae*, *A. pterocarpi*, and *A. schimae* differ from previously known *Amphisphaeria* species in Amphisphaeriaceae. This distinction is supported by combined analyses using maximum likelihood and Bayesian inference of nuclear ribosomal large subunit rDNA (LSU) and the internal transcribed spacer (ITS) sequence matrix. *Amphisphaeria mesuae* can be distinguished from *A. ailaoshanensis* by the presence of larger ascomata, smaller asci, ellipsoidal ascospores, and the absence of a sheath surrounding the ascospores. *Amphisphaeria mimusopis* differs from its phylogenetically related sister taxon by possessing smaller asci, larger ascospores, and narrower paraphyses. *Amphisphaeria paraserianthis* differs from closely related taxa in its ostiolate ascomata and 3-septate ascospores. *Amphisphaeria pterocarpi* can be distinguished from the taxon with which it clusters by having smaller ascomata and ascospores. *Amphisphaeria pseudomicheliae* has larger ascomata and asci, but smaller ascospores, compared to its sister taxon, *A. micheliae*. *Amphisphaeria schimae* differs from closely related taxa in its larger ascomata, asci, and ascospores. *Amphisphaeria micheliae* was also collected here and is reported as a new record on *Senna siamea*. This study contributes to the expansion of the taxonomic framework of *Amphisphaeria*.

## ﻿Introduction

Amphisphaeriales was described by Eriksson and Hawksworth in 1986 and is phylogenetically closely related to Xylariales within the Xylariomycetidae group (Senanayake et al. 2015; Samarakoon et al. 2022). The type genus *Amphisphaeria* was established by Cesati et al. in 1863 for the family Amphisphaeriaceae, and two additional genera, *Griphosphaerioma* and *Lepteutypa*, were later added to the family (Wijayawardene et al. 2018). However, *Lepteutypa* was ultimately synonymized with *Amphisphaeria* based on holomorphic morphology and multigene phylogeny by Samarakoon et al. (2020), while *Griphosphaerioma* was regarded as a synonym of *Labridella* by Rossman et al. (2016). Currently, Amphisphaeriaceae includes *Amphisphaeria* and *Labridella* (Hyde et al. 2024a).

*Amphisphaeria*, typified by *A. umbrina*, generally comprises immersed, clypeate, and ostiolate ascomata that appear as elevated, blackened, round spots on the host surface (Samarakoon et al. 2020). The peridium layers comprise a hyaline inner layer and a brown outer layer of cells. Paraphyses are filamentous, septate, and flexuous (Wang et al. 2004). The cylindrical asci are 8-spored, with J+ or J-, discoid, tubular, or wedge-shaped apical rings, and the ascospores are 1–3 septate, ellipsoidal, and brown (Cesati and De 1863; Wang et al. 2004; Samarakoon et al. 2019, 2020, Samarakoon 2023). Both coelomycetous and hyphomycetous asexual morphs are reported in *Amphisphaeria* (Samarakoon et al. 2020). Saprobic *Amphisphaeria* species have been reported from woody branches and various monocotyledon hosts, including grasses from different geographical regions (Samarakoon et al. 2019). Members of *Amphisphaeria* are predominantly found on dead plant materials in both terrestrial and marine habitats (Senanayake et al. 2015, 2019; Samarakoon et al. 2019; Sun et al. 2025). Given the widespread nature and diverse lifestyles of *Amphisphaeria* species, further taxonomic studies are essential.

In this study, we examine the taxonomy, lifestyle, ecological roles, and distribution of *Amphisphaeria* species in northern Thailand. We introduce six species of *Amphisphaeria*, along with a new host record. These taxa were isolated from dead twigs of *Mesua* sp., *Mimusops elengi*, *Paraserianthes lophantha*, *Pterocarpus* sp., *Schima wallichii*, *Senna siamea*, and an undetermined host, from two provinces in northern Thailand. Morphological illustrations of the taxa are provided. Phylogenetic studies incorporating combined nuclear ribosomal large subunit rDNA (LSU) and the internal transcribed spacer (ITS) region confirm the taxonomic placements of these species as novel within *Amphisphaeria* and also support the finding of a new host record of an existing *Amphisphaeria* taxon.

## ﻿Materials and methods

### ﻿Sample collection, isolation, and morphology

Dead twigs were collected from the premises of the Mushroom Research Center (MRC) and Mae Fah Luang University during the cold (October, November) and wet (July) seasons in northern Thailand. After the collection details were recorded (Rathnayaka et al. 2025), specimens were brought to the laboratory in plastic bags and stored in a paper envelope. A Motic SMZ 168 Series stereo microscope (Leica Microsystems Company, Germany) was used to observe the fungi colonizing the host, and water-mounted slides were prepared to examine their micro-morphological characters. Melzer’s reagent and Indian ink were utilized for further morphological investigations. Digital images of the micro morphological features were captured using a Canon 750D camera (Canon, Tokyo, Japan) mounted on a Nikon ECLIPSE E600 compound microscope (Nikon, Tokyo, Japan), with objective lenses providing magnifications of 10×, 20×, 40×, 60×, and 100×. Photo plates were produced using Adobe Photoshop CS6 software (Adobe Systems, USA). Measurements of the fungal characteristics were conducted using the Tarosoft® Image Framework software (version 0.9.7).

Single spore isolations were performed as outlined by Senanayake et al. (2020) to obtain pure cultures. Germinated spores were identified after 24 hours of growth on malt extract agar (MEA), then transferred to fresh MEA media and incubated at 25 °C. Pure cultures were maintained on malt extract agar (MEA) at 25 °C, and their cultural characteristics were observed after one month of growth.

Herbarium materials and cultures were deposited in the Mae Fah Luang University Herbarium (MFLU) and Mae Fah Luang University Culture Collection (MFLUCC), respectively. Index Fungorum and faces of fungi numbers were obtained (Jayasiri et al. 2015; Index Fungorum 2025). The species descriptions were added to the Greater Mekong Subregion database (https://gmsmicrofungi.org/) (Chaiwan et al. 2021) and the Fungalpedia webpage (Hyde et al. 2023).

### ﻿DNA extraction, PCR amplification, and sequencing

Genomic DNA was extracted from fresh mycelia grown on MEA for 15 days, or DNA was extracted directly from the fruiting bodies using a DNA Extraction Kit (Omega Biotek) following the manufacturer’s protocol. The polymerase chain reaction (PCR) was conducted in a total volume of 25 μL, comprising 12.5 μL of 2× Power TaqPCR Master Mix, 1 μL of each primer (20 M), 2 μL of genomic DNA, and 8.5 μL of distilled water. PCR was performed using an Eppendorf thermal cycler (Mastercycler X50s) to amplify the LSU and ITS loci under the conditions outlined in Table 1. Agarose gel electrophoresis was performed to assess the quality of PCR products prior to sequencing at SolGent Co., South Korea.

**Table 1.** Primers and adapted PCR conditions applied for individual locus.

Gene region	Primer pairs	PCR conditions	References
LSU	LR0R/LR5	95 °C/30 s, 55 °C/50 s, 72 °C/60 s	Vilgalys and Hester (1999)
ITS	ITS5/ITS4	95 °C/30 s, 55 °C/50 s, 72 °C/60 s	White et al. (1990)

The PCR thermal cycling protocol consists of an initial step at 95 °C for 5 minutes, followed by a final elongation step at 72 °C for 10 minutes, and concluding with a hold at 4 °C, as well as annealing at 55 °C.

### ﻿Phylogenetic analyses

SeqMan (DNAStar, Inc., Madison, WI, USA) was used to generate consensus sequences from the forward and reverse chromatograms obtained. The sequences were subsequently subjected to a BLASTn search in NCBI (https://blast.ncbi.nlm.nih.gov/). The LSU and ITS sequences for *Amphisphaeria* species were retrieved from the GenBank database (Table 2). Each gene locus was aligned using the default settings in MAFFT v. 7 (https://mafft.cbrc.jp/alignment/server/) (Katoh et al. 2019) and trimmed with trimAl v. 1.2 (Capella-Gutiérrez et al. 2009). Single locus alignments were concatenated using BioEdit v. 7.0.5.2 (Hall 1999). ALTER (http://www.sing-group.org/ALTER/) was used to convert FASTA files into PHYLIP format. Single-locus and multi-locus aligned datasets were analyzed separately using maximum likelihood (ML) and Bayesian inference (BI). Maximum likelihood analysis was performed using IQ webserver (http://iqtree.cibiv.univie.ac.at/) with bootstrap support for 1000 replicates (Nguyen et al. 2015).

**Table 2.** Taxa used in the phylogenetic analyses and their corresponding GenBank accession numbers. Sequences obtained in this study are in bold.

Taxon	Strain	LSU	ITS	Reference
*Amphisphaeria acericola*	MFLU 16-2479*	MK640424	MK640423	Senanayake et al. (2019)
*Amphisphaeria acericola*	MFLUCC 14-0842*	MF614131	MF614128	Senanayake et al. (2019)
*Amphisphaeria ailaoshanensis*	KUNCC 23-15521	PP584771	PP584674	Dissanayake et al. (2024)
*Amphisphaeria ailaoshanensis*	KUNCC 23-15520*	PP584770	PP584673	Dissanayake et al. (2024)
*Amphisphaeria camelliae*	HKAS 107021*	MT756615	MT756621	Samarakoon et al. (2020)
*Amphisphaeria camelliae*	MFLU 20-0181*	MT756616	MT756622	Samarakoon et al. (2020)
*Amphisphaeria chiangmaiensis*	CMUB 40017*	OR507152	OR507139	Samarakoon (2023)
*Amphisphaeria chiangmaiensis*	MFLU 23-0411*	OR507153	OR507140	Samarakoon (2023)
*Amphisphaeria curvaticonidia*	MFLU 18-0789*	MT756617	MT756623	Samarakoon et al. (2020)
*Amphisphaeria curvaticonidia*	HKAS 102288*	MT756618	MT756624	Samarakoon et al. (2020)
*Amphisphaeria falcata*	CGMCC3.23740*	OQ645284	OQ645270	Chang et al. (2025)
*Amphisphaeria ﬂava*	MFLU 18-0102*	MH971234	MH971224	Samarakoon et al. (2019)
*Amphisphaeria fuckelii*	WU 33555	N/A	KT949903	Jaklitsch et al. (2016)
*Amphisphaeria fuckelii*	CBS 140409*	N/A	KT949902	Jaklitsch et al. (2016); Voglmayr et al. (2019)
*Amphisphaeria guttulata*	MFLUCC 22-0052	N/A	NR_190966	Liu et al. (2024b)
*Amphisphaeria guttulata*	MFLUCC 22-0078	OQ101583	OQ101582	Liu et al. (2024b)
*Amphisphaeria hibiscicola*	HKAS 136910	PQ570865	PQ570847	Sun et al. (2025)
*Amphisphaeria hongheensis*	MHZU 24-0515	PQ166524	PQ165968	Liu et al. (2024a)
*Amphisphaeria hydei*	CMUB 40016*	OR507154	OR507141	Samarakoon (2023)
*Amphisphaeria hydei*	MFLU 23-0412*	OR507155	OR507142	Samarakoon (2023)
*Amphisphaeria karsti*	GZAAS 20-0147*	OR209622	OR224991	Zhang et al. (2023)
*Amphisphaeria karsti*	GZAAS 20-148	OR209623	OR224992	Zhang et al. (2023)
*Amphisphaeria kunmingensis*	KUNCC 23-15522*	PP584772	PP584675	Dissanayake et al. (2024)
*Amphisphaeria kunmingensis*	KUNCC 23-15523	PP584773	PP584676	Dissanayake et al. (2024)
*Amphisphaeria magna*	HKAS 130271	PP584775	PP584678	Dissanayake et al. (2024)
*Amphisphaeria magna*	HKAS 130270*	PP584774	PP584677	Dissanayake et al. (2024)
*Amphisphaeria mangrovi*	PUFD37	MG844275	MG844283	Dissanayake et al. (2024)
***Amphisphaeria mesuae***	MFLUCC 25-0197*	**PV299568**	**PV393830**	**(This study)**
*Amphisphaeria micheliae*	UESTCC 23.0125	OR253277	OR253118	Li et al. (2024)
*Amphisphaeria micheliae*	UESTCC 23.0123	OR253249	OR253097	Li et al. (2024)
*Amphisphaeria micheliae*	UESTCC 23.0124	OR253280	OR253121	Li et al. (2024)
*Amphisphaeria micheliae*	HKAS 107012*	MT756619	MT756625	Samarakoon et al. (2020)
*Amphisphaeria micheliae*	MFLUCC 24-0324	PQ340163	PQ340156	Pathirana et al. (2025)
*Amphisphaeria micheliae*	MFLU 20-0172*	MT756620	MT756626	Samarakoon et al. (2020)
*Amphisphaeria micheliae*	MFLU 21-0207	OK179729	OK284457	De Silva et al. (2022)
***Amphisphaeria micheliae***	**MFLU 25-077**	**PV299571**	**PV290310**	**(This study)**
***Amphisphaeria mimusopis***	MFLUC 25-0076*	**PV299569**	**PV366837**	**(This study)**
***Amphisphaeria mimusopis***	**MFLU 25-0159**	**N/A**	**PV522822**	**(This study)**
*Amphisphaeria neoaquatica*	MFLUCC 14-0045*	MK835805	MK828607	Luo et al. (2019)
*Amphisphaeria oleae*	UESTCC: 23.0120	OR253314	OR253157	Li et al. (2024)
*Amphisphaeria oleae*	CGMCC: 3.24959*	OR253313	OR253156	Li et al. (2024)
*Amphisphaeria orixae*	GZCC 22-2031*	OQ064543	OQ064541	Wang et al. (2023)
*Amphisphaeria orixae*	GZCC 22-2032*	OQ064544	OQ064542	Wang et al. (2023)
***Amphisphaeria paraserianthis***	MFLU 25-0075*	**PV393833**	**PV393832**	**(This study)**
*Amphisphaeria parvispora*	MFLU 18-0767*	MW240574	MW240644	Samarakoon et al. (2022)
***Amphisphaeria pseudomicheliae***	MFLU 25-0074*	**PV299570**	**PV393834**	**(This study)**
***Amphisphaeria pterocarpi***	**MFLU 25-0073**	**PV299564**	**PV366837**	**(This study)**
***Amphisphaeria pterocarpi***	MFLUCC 25-0195*	**PV299566**	**PV366836**	**(This study)**
*Amphisphaeria qujingensis*	KUMCC 19-0187*	MN556316	MN477033	Dissanayake et al. (2020)
*Amphisphaeria qujingensis*	KUMCC 19-0186*	MN707566	MN707568	Dissanayake et al. (2020)
*Amphisphaeria sambuci*	WU 33557	N/A	KT949905	Jaklitsch et al. (2016)
*Amphisphaeria sambuci*	CBS 131707*	NG_066215	KT949904	Jaklitsch et al. (2016), Liu et al. (2019)
***Amphisphaeria schimae***	**MFLUCC 25-0196** *	**PV299567**	**PX488288**	**(This study)**
***Amphisphaeria schimae***	**MFLU 25-0071**	**PV299565**	**PX488290**	**(This study)**
*Amphisphaeria shangrilaensis*	HKAS 130273	PP584776	PP584679	Dissanayake et al. (2024)
*Amphisphaeria shangrilaensis*	HKAS 130272*	PP584777	PP584680	Dissanayake et al. (2024)
*Amphisphaeria sorbi*	MFLUCC 13-0721*	KP744475	KR092797	Liu et al. (2015)
*Amphisphaeria* sp.	KoLRI 053241	N/A	MZ855365	Yang et al. (2022)
*Amphisphaeria thailandica*	MFLU 18-0794*	MH971235	MH971225	Samarakoon et al. (2019)
*Amphisphaeria umbrina*	HKUCC 994	AF452029	AF009805	Jeewon and Hyde (2003), Jaklitsch et al. (2016)
*Amphisphaeria umbrina*	PRA-JV24328	N/A	OL396664	Vondrák et al. (2022)
*Amphisphaeria uniseptata*	CBS 114967*	MH554197	MH553979	Liu et al. (2019)
*Amphisphaeria verniciae*	UESTCC: 23.0122	OR253270	OR253155	Samarakoon (2023)
*Amphisphaeria verniciae*	CGMCC: 3.24960*	OR253269	OR253154	Samarakoon (2023)
*Amphisphaeria xishuangbannaense*	KUNCC 23-15525	PP584779	PP584682	Dissanayake et al. (2024)
*Amphisphaeria xishuangbannaense*	KUNCC 23-15524*	PP584778	PP584681	Dissanayake et al. (2024)
*Amphisphaeria yunnanensis*	KUMCC 19-0189*	MN550992	MN550997	Dissanayake et al. (2020)
*Amphisphaeria yunnanensis*	KUMCC 19-0188*	MN556306	MN477177	Dissanayake et al. (2020)
*Beltrania rhombica*	CBS 123.58*	MH869260	MH857718	Liu et al. (2019); Vu et al. (2019)
*Beltraniella endiandrae*	CBS 137976*	KJ869185	KJ869128	Crous et al. (2014)
*Beltraniopsis longiconidiophora*	MFLUCC 17-2139*	MF580256	MF580249	Lin et al. (2017)
*Neoarthrinium moseri*	CBS 164.80*	LN851049	LN850995	Sandoval-Denis et al. (2016)
*Neoarthrinium trachycarpi*	CFCC 53039*	N/A	MK301099	Yan et al. (2019)
*Pidoplitchkoviella terricola*	CBS 180.77*	AF096197	MH861046	Suh and Blackwell (1999), Vu et al. (2019)

MrModeltest v. 2.2 was used to estimate the evolution model using the Akaike information criterion (AIC), implemented in PAUP v. 4.0b10 (Nylander 2004). MrBayes v. 3.1.2 was used to conduct Bayesian inference (BI) analyses for estimating posterior probabilities (PP) through Markov chain Monte Carlo sampling (MCMC) under the GTR+I+G model (Huelsenbeck et al. 2001; Ronquist and Huelsenbeck 2003). Markov chains were executed for 1,000,000 generations, with trees sampled every 100^th^ generation. The initial 25% of trees were discarded during the burn-in phase, while the remaining trees were used to compute the posterior probability (PP) in the majority rule consensus tree. FigTree v.1.4 was employed to visualize the phylograms (Rambaut and Drummond 2012), which were subsequently edited using Microsoft PowerPoint.

Abbreviations: CBS: Westerdijk Fungal Biodiversity Institute, Utrecht, the Netherlands; CFCC: China Forestry Culture Collection Center, Research Institute of Forest Ecology, Environment and Protection, Beijing, China; CGMCC: China General Microbiological Culture Collection Center, Beijing, China; CMUB: Chiang Mai University, Chiang Mai, Thailand; GZAAS and GZCC: Guizhou Academy of Agricultural Sciences, Guizhou, China; HKAS: Herbarium of Cryptogams Kunming Institute of Botany Academia Sinica, China; HKUCC: University of Hong Kong Culture Collection, Department of Ecology and Biodiversity, Hong Kong, China; KUMCC: Kunming Institute of Botany Culture Collection, China; KoLRI: Kholodny Institute of Botany, Tereshchenkivska, Kiev, Ukraine; MFLU, MFLUCC: Mae Fah Luang University, Chiang Rai, Thailand; PRA: The Herbarium of the Institute of Botany, Czech Academy of Sciences, Průhonice, Czech Republic; UESTCC: University of Electronic Science and Technology culture Collection, Xiyuan, Chengdu, China; WU: The Herbarium of the University of Vienna, Austria. Type species are denoted in ‘*’; “N/A” indicates the sequences are not available.

## ﻿Results

### ﻿Sequence alignment and phylogenetic analyses

The combined LSU and ITS sequence matrix comprised 74 Amphisphaeriaceae taxa, including our ten new strains. The tree is rooted with *Beltrania rhombica* (CBS 123.58), *Beltraniella endiandrae* (CBS 137976), and *Beltraniopsis longiconidiophora* (MFLUCC 17-2139). The combined alignment comprised 1976 characters, including gaps (LSU: 1–1,332 and ITS: 1,333–1,970). Both ML and BI analyses yielded trees with similar topologies.

**Figure 1.**
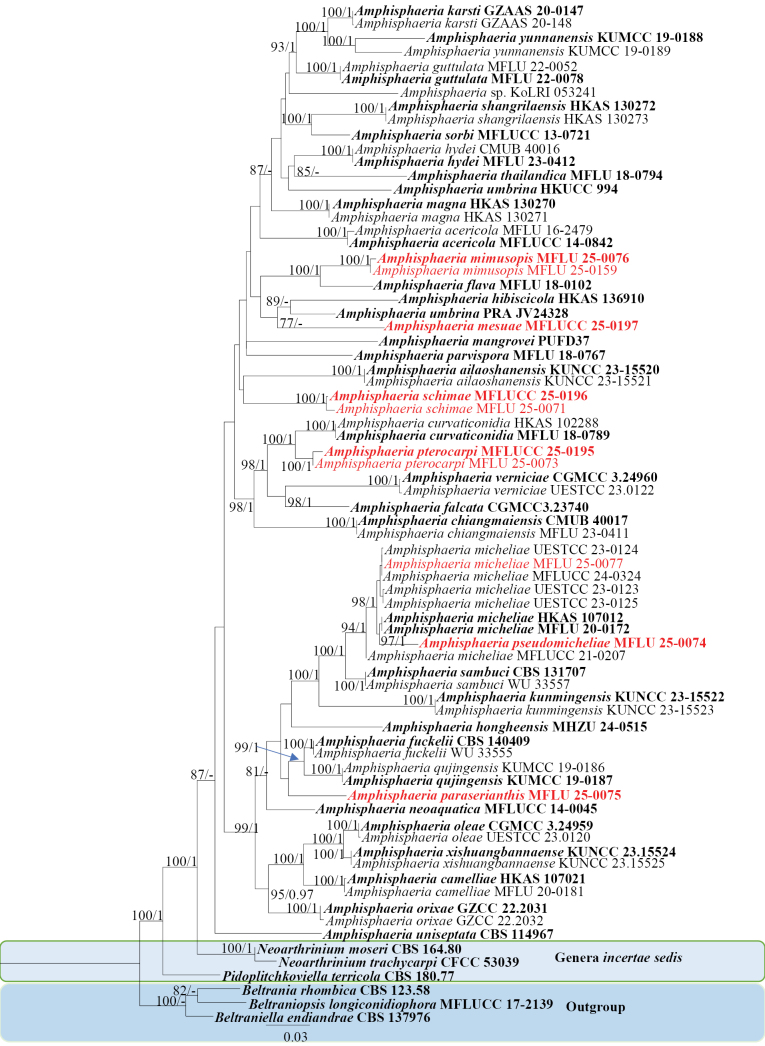
Phylogram generated from ML analysis based on combined LSU, ITS sequence data. Bootstrap support values for ML ≥75% and PP ≥0.90 are indicated near the corresponding nodes. The tree is rooted with *Beltrania rhombica* (CBS123.58), *Beltraniella endiandrae* (CBS:137976), and *Beltraniopsis longiconidiophora* (MFLUCC17-2139). Type and reference strains are in bold, while the novel strains are in bold red.

The ML phylogram was used as the backbone tree (Fig. 1). The best-scoring RAxML tree had an optimization likelihood value of -14759.462. The matrix contained 1,000 distinct patterns, with 29.25% of the characters being undetermined or gaps. Estimated base frequencies were: A = 0.250, C = 0.250, G = 0.250, T = 0.250; substitution rates AC = 0.72949, AG = 2.67549, AT = 1.00000, CG = 0.72949, CT = 3.60004, GT = 1.0; the gamma distribution shape parameter was 0.467, Tree Length: 2.512. For the Bayesian analysis, the best-fit models generated from MrModeltest under the Akaike information criterion (AIC) are as follows: LSU: TI M3+I+G and ITS: TVM+I+G.

*Amphisphaeria mesuae* (MFLUCC 25-0197) clustered with *Amphisphaeria hibiscicola* (HKAS 136910) and *Amphisphaeria umbrina* (PRA JV24328). *Amphisphaeria micheliae* (MFLU 25-0077) clustered in a clade shared by other *A. micheliae* strains (HKAS 107012, MFLU 20-0172, MFLUCC 21-0207, MFLUCC 24-0324, UESTCC 23-0123, UESTCC 23-0124, UESTCC 23-0125). *Amphisphaeria mimusopis* (MFLU 25-0076, MFLU 25-0159) was sister to *A. flava* (MFLU 18-0102). *Amphisphaeria paraserianthis* (MFLU 25-0075) formed a separate lineage, basal to *A. neoaquatica* (MFLUCC 14-0045) and *A. hongheensis* (GMB1135). *Amphisphaeria pseudomicheliae* was a sister to the *A. micheliae* group. *Amphisphaeria pterocarpi* (MFLU 25-0073, MFLUCC 25-0195) formed a separate lineage, sister to *A. curvaticonidia* (MFLUCC 18-0620, HKAS 102288). *Amphisphaeria schimae* (MFLU 25-0071, MFLUCC 25-0196) is sister to *A. ailaoshanensis* (KUNCC 23-15520, KUNCC 23-15521).

### ﻿Taxonomy

#### ﻿ Amphisphaeria mesuae

Taxon classificationFungiAmphisphaerialesAmphisphaeriaceae

Z.L. Tun & K.D. Hyde sp. nov.

45E59076-317D-5F4D-AB3B-FFD94A4465BE

Index Fungorum: IF903423

Facesoffungi Number: FoF17659

Fig. 2

##### Etymology.

The epithet refers to the host genus, *Mesua*, from which the fungus was isolated.

**Figure 2.**
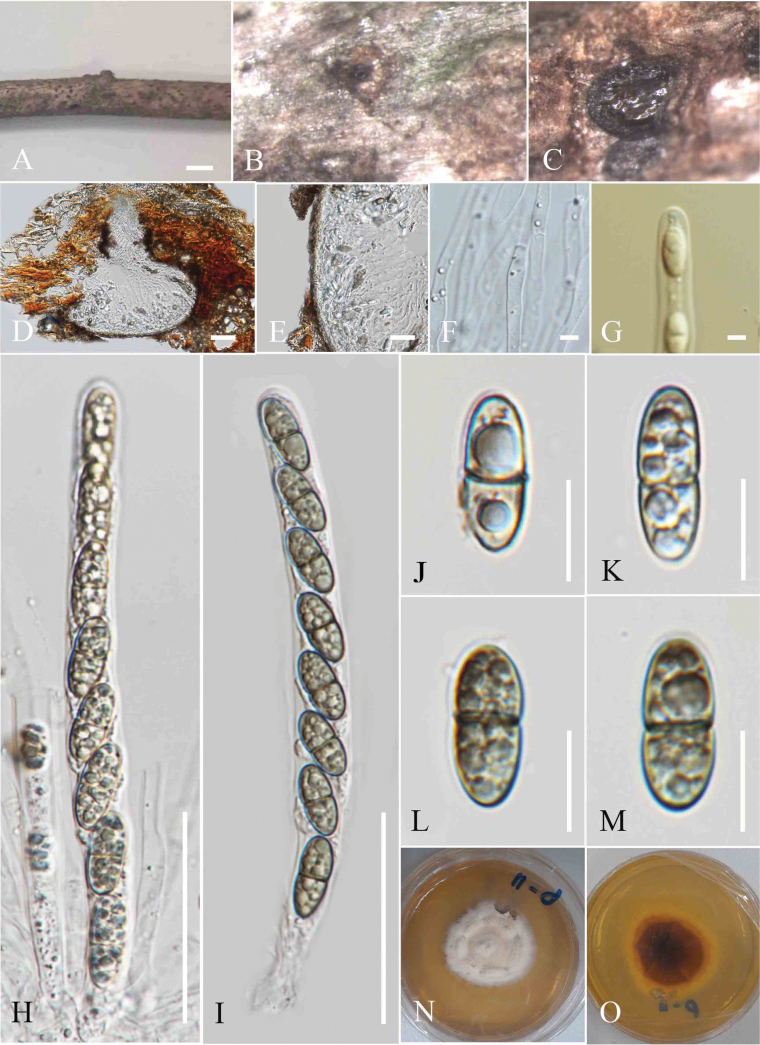
*Amphisphaeria mesuae* (MFLU 25-0069, holotype). A. Decaying woody twig; B. Appearance of an ascoma on the host; C, D. Vertical section through an ascoma; E. Vertical section of peridium; F. Paraphyses; G. J+ Apical ring; H, I. Asci; J–M. Ascospores; N. Upper view of culture; O. Reverse view of culture. Scale bars: 200 μm (B, C); 100 μm (D); 20 μm (E); 5 μm (F, G); 40 μm (H, I); 10 μm (J–M).

##### Holotype.

MFLU 25-0069.

##### Description.

***Saprobic*** on decaying twigs of *Mesua* sp. **Sexual morph**: ***Ascomata*** 520–580 μm high, 260–290 µm wide (xˉ = 549 × 272 µm, n = 5), immersed, visible as black spots with tiny pores, flat, scattered or aggregated, globose to subglobose, ostiolate. ***Peridium*** 14–18 µm (xˉ = 15 µm, n = 5), two-layered; outer layer wide, comprising thick-walled, dark brown cells of ***textura angularis***, inner layer thin, composed of hyaline ***textura angularis*** cells. ***Paraphyses*** 3–5 µm wide, hyaline, septate, longer than asci, narrow towards the apex. ***Asci*** 86–134 × 7–13 µm (xˉ = 115 × 9 µm, n = 20), 8-spored, unitunicate, cylindrical, with short pedicel, apically rounded, with a J+, apical ring. ***Ascospores*** 14–18 × 5–6.8 µm (xˉ = 15 × 6 µm, n = 20), uniseriate, ellipsoidal, hyaline when immature, turning sub-hyaline to pale brown at maturity, 1-septate, slightly constricted at the septum, guttulate, smooth-walled, lacking a sheath in Indian ink. **Asexual morph**: Not observed.

##### Culture characteristics.

Colonies on MEA, reaching 4 cm diam. after 15 days at 27 °C, from above white to pale yellow radiating outwards, dense, circular to slightly irregular, flattened with smooth surface, with smooth margin; reverse pale brown in the middle, yellowish brown at the margin.

##### Material examined.

Thailand • Chiang Rai Province, Mae Fah Luang University (20°02′42″N, 99°53′41″E), on decaying dead twigs of *Mesua* sp. (Calophyllaceae), 02 October 2023, Zaw Lin Tun P11 (holotype MFLU 25-0069); ex-type culture MFLUCC 25-0197.

##### Notes.

Phylogenetic analyses revealed that *Amphisphaeria mesuae* (MFLUCC 25-0197) clustered with *Amphisphaeria hibiscicola* (HKAS 136910) and *Amphisphaeria umbrina* (PRA JV24328) in (Fig. 1). The interspecies genetic distances between *A. mesuae* (MFLU25- 0069) and *A. hibiscicola* (HKAS 136910) showed the following base pair differences (without gaps): 5.7% for LSU (49/935 bp) and 11.18% for ITS (66/559 bp). *Amphisphaeria mesuae* (MFLUCC 25-0197) and *Amphisphaeria umbrina* (PRA JV24328) showed the following base pair differences (without gaps): 3.3% for LSU (28/844 bp) and 9.45% for ITS (54/571 bp). Morphologically, *A. mesuae* differs from *A. hibiscicola* by having taller ascomata (520–580 μm) compared to those of *A. hibiscicola* (212–450 μm), whereas *A. hibiscicola* has broader ascomata (456–570 μm wide) than *A. mesuae* (260–290 μm) (Sun et al. 2025). The asci of *A. mesuae* (86–134 × 7–13 µm) are smaller than those of *A. hibiscicola* (122–152 × 6–8 µm) (Sun et al. 2025). The ascospores of *A. mesuae* (14–18 × 5–6.8 µm) are ellipsoidal, while the ascospores of *A. hibiscicola* (8–14 × 3.5–5 μm) are fusiform (Sun et al. 2025). *Amphisphaeria hibiscicola* has a gelatinous sheath, whereas *A. mesuae* lacks a sheath (Sun et al. 2025

Morphologically, *A. mesuae* differs from *A. umbrina* by having higher ascomata (520–580 μm vs. 400–480 μm) but narrower width (260–290 μm vs. 560–640 μm) (Wang et al. 2004).The asci of *A. mesuae* (122–152 × 6–8 µm) are also smaller than those of *A. umbrina* (150–170 × 11–13 µm) (Wang et al. 2004). Similarly, the ascospores of *A. mesuae* (14–18 × 11–12 µm) are smaller than those of *A. umbrina* (18–22 × 6–8 μm) (Wang et al. 2004).

Based on the distinct morphology and phylogenetic evidence, along with the recommendations for species delineation proposed by Chethana et al. (2021) and Maharachchikumbura et al. (2021), we introduce *A. mesuae* as a new species.

#### ﻿ Amphisphaeria micheliae

Taxon classificationFungiAmphisphaerialesAmphisphaeriaceae

Samarak., Jian K. Liu & K.D. Hyde, 2020

5A9ED485-F938-5CFD-9E3D-2586DC1E0AF2

MycoBank No: 836112

Facesoffungi Number: FoF08752

Fig. 3

##### Description.

***Saprobic*** on dead twigs of *Senna siamea*. **Sexual morph**: ***Ascomata*** 308–320 µm high, 348–360 µm wide, (xˉ = 312 × 353 µm, n = 5), immersed, visible as black spots in light-coloured areas on the host, solitary, scattered, subglobose to oblate, papillate. ***Ostiole*** 100–106 µm high, 65–70 µm diam (xˉ = 102 × 67 µm, n = 5), centric. ***Peridium*** 45–58 µm (xˉ = 48 µm, n = 5) two-layered; outer layer thick, dense, made up of red-dish-brown cells of ***textura angularis***; inner layer thin, comprising hyaline cells. ***Paraphyses*** 3–5 µm wide (xˉ = 4.4 µm, n = 5), hyaline, longer than asci, septate, guttulate, embedded in a gelatinous matrix. ***Asci*** 85–115 × 6.5–7 µm (xˉ = 101 × 6.8 µm, n = 20), 8-spored, unitunicate, cylindrical, thin-walled, short-pedicellate, apically rounded, with a J+, discoid apical ring. ***Ascospores*** 15–16 × 5–6 µm (xˉ = 15.5 × 5.9 µm, n = 20), uniseriate, oblong or narrowly fusiform, guttulate, hyaline when immature, turning sub-hyaline to olivaceous grey, 1-septate, slightly constricted at the septum, straight to slightly curved, smooth-walled, lacking a sheath in Indian ink. **Asexual morph**: Not observed.

**Figure 3.**
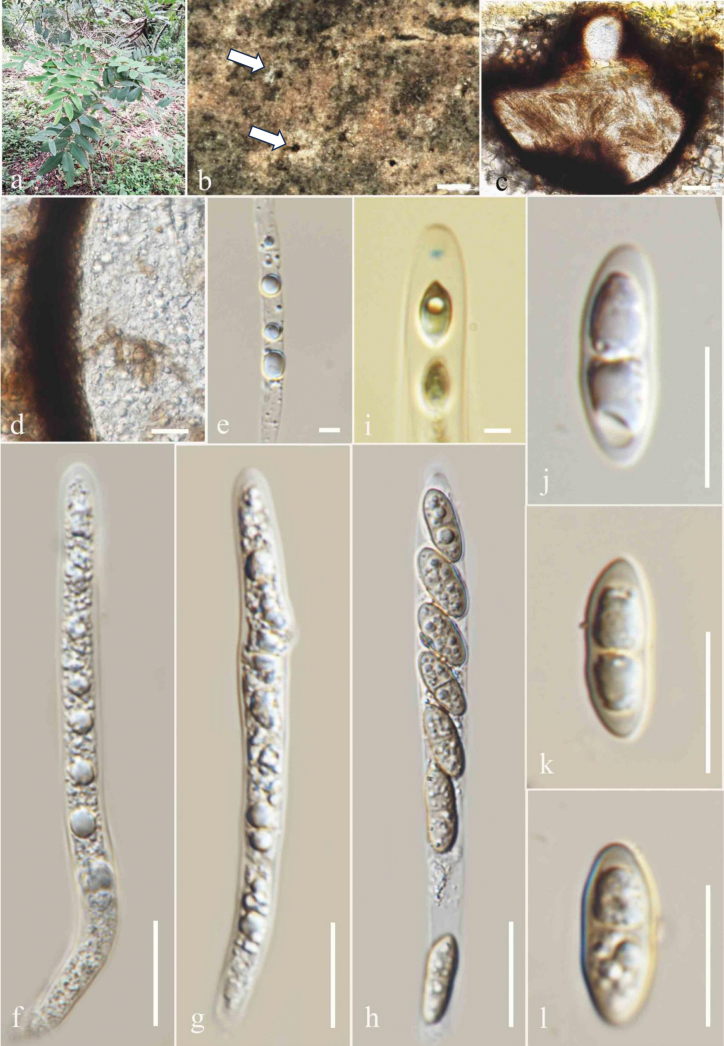
*Amphisphaeria micheliae* (MFLU 25-0077). A. *Senna siamea* tree; B. Appearance of ascomata on host (arrows indicate ascomata visible as black spots); C. Vertical section through an ascoma; D. Vertical section of ascomatal wall; E. Paraphyses; F–H. Asci; I. J+ Apical ring; J–L. Ascospores. Scale bars: 200 μm (C); 100 μm (D); 20 μm (E); 5 μm (F, G); 50 μm (H, I); 10 μm (J–L).

##### Material examined.

Thailand • Chiang Rai Province, Mae Fah Luang University premises (20°02′42″N 99°53′41″E), on dead decaying twigs of *Senna siamea* (Fabaceae), 02 October 2024, Zaw Lin Tun T7, (MFLU 25-0077).

##### Notes.

Based on the phylogenetic analyses, our strain (MFLU 25-0077) clustered with *Amphisphaeria micheliae* group (Fig. 1). Morphologically, our strain (MFLU25-0077) resembles the type of *A. micheliae* (MFLU 20-0172) (Samarakoon et al. 2020). The asci length of our collection (MFLU25-0077) measures 85–115 × 6.5–7 µm, while those of the type of *A. micheliae* (HKAS 107012) range from 92–135 × 7–10.5 µm (Samarakoon et al. 2020). The ascospore length of *A. micheliae* (MFLU 25-0077 and HKAS 107012) is also similar (15–16 × 5–6 µm vs. 15.5–21 × 6–7.5 µm) (Samarakoon et al. 2020). The comparison of inter-species genetic distances between our strain (MFLU25-0077) and HKAS 107012 reveals largely similar base pair differences in the LSU (99%) and ITS (99%) regions. Based on this morpho-phylogenetic evidence, we identify our isolate as *A. micheliae*. Previously, *A. micheliae* was isolated from dead twigs of *Acer truncatum*, *Alstonia scholaris*, *Micromelum integerrimum*, and *Michelia alba* in China and Thailand (Samarakoon et al. 2020; De Silva et al. 2022; Li et al. 2024; Pathirana et al. 2025). This is the first report of *A. micheliae* from *Senna siamea* in Thailand.

#### ﻿ Amphisphaeria mimusopis

Taxon classificationFungiAmphisphaerialesAmphisphaeriaceae

Z.L. Tun & K.D. Hyde sp. nov.

CED7A44E-2ADA-5329-A2E4-BE2F6DCA0AD8

Index Fungorum: IF903593

Facesoffungi Number: FoF17660

Fig. 4

##### Etymology.

The epithet refers to the host genus, *Mimusops*, from which the fungus was isolated.

**Figure 4.**
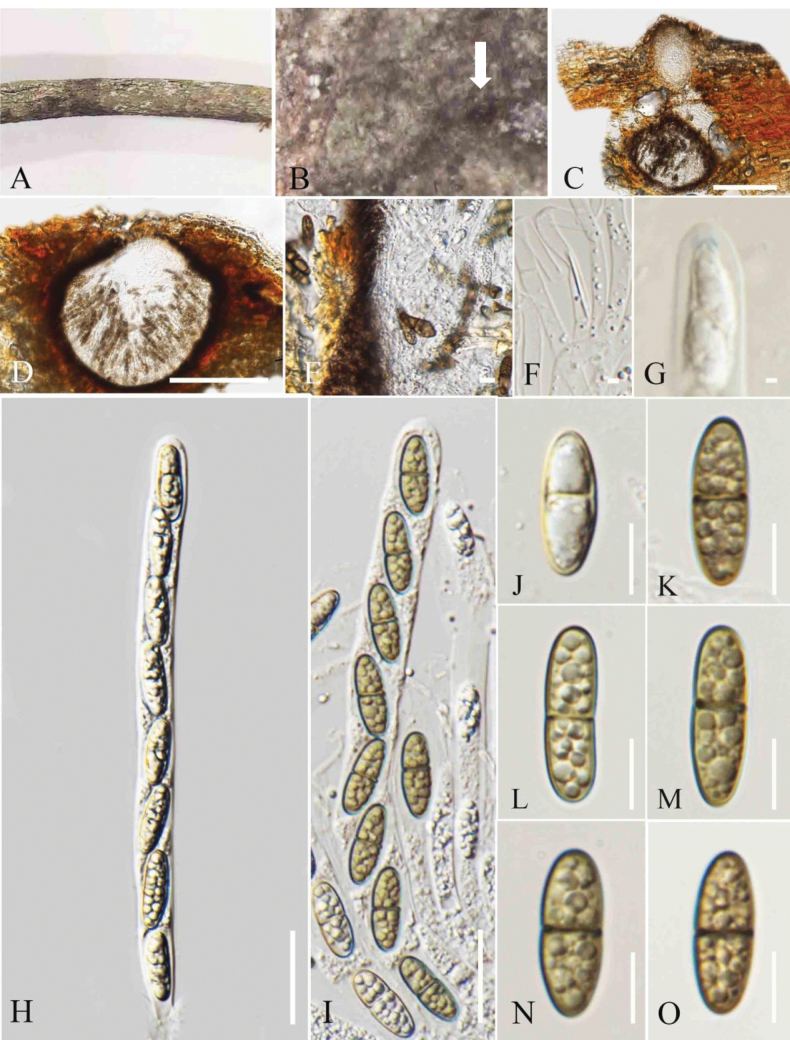
*Amphisphaeria mimusopis* (MFLU 25-0076, holotype). A. Decaying dead branch; B. Appearance of ascomata on host (arrows indicate the ostiolar openings); C, D. Vertical section through an ascoma; E. Vertical section of ascomatal wall; F. Paraphyses; G. J+ Apical ring; H, I. Asci; J–O. Ascospores. Scale bars: 500 μm (C); 200 μm (D); 10 μm (E); 5 μm (F, G); 50 μm (H, I); 20 μm (J–O).

##### Holotype.

MFLU 25-0076.

##### Description.

***Saprobic*** on the decaying branch of *Mimusops elengi*. **Sexual morph**: ***Ascomata*** 286–370 μm high, 364–455 µm wide, (xˉ = 321 × 397 µm, n = 5), immersed, visible as black spots with tiny pores, staining the host surface pale reddish brown around the ostioles, solitary, scattered to aggregated, globose to sub-globose. ***Ostiole*** central, prominent, 150–166 μm high, 82–84 µm wide (xˉ = 154 × 82 µm, n = 5). ***Peridium*** 12–25 µm (xˉ = 20 µm, n = 5), two-layered; outer layer thick, dense, reddish-brown cells of ***textura angularis***, inner layer thin, cells hyaline, of ***textura angularis***. ***Paraphyses*** 3–6 µm wide (xˉ = 4.2 µm, n = 5), hyaline, septate, longer than asci and embedded in a gelatinous matrix. ***Asci*** 126–164 × 8–13 µm (xˉ = 145.7 × 10. 2 µm, n = 20), 8-spored, unitunicate, cylindrical, with short pedicel, apically rounded, with a J+, discoid, apical ring. ***Ascospores*** 18–22 × 5–7 µm (xˉ = 20 × 6 µm, n = 20), uniseriate, cylindrical to oblong, hyaline when immature, turning subhyaline to brown at maturity, 1-septate, slightly constricted at the septum, guttulate, smooth-walled, lacking a sheath in Indian ink. **Asexual morph**: Not observed.

##### Material examined.

Thailand • Chiang Rai Province, Mae Fah Luang University (20°02′42″N, 99°53′41″E), on the dead, decaying twigs of *Mimusops elengi* (Sapotaceae), 2 October 2023, ZL Tun P3 (holotype MFLU 25-0076).

##### Additional specimens examined.

Thailand • Chiang Rai Province, Mae Fah Luang University (20°02′42″N, 99°53′41″E), on the dead decaying branch of *Mimusops elengi* (Sapotaceae), 2 October 2023, ZL Tun P3A (MFLU 25-0159).

##### Notes.

*Amphisphaeria mimusopis* is sister to *A. flava* (MFLU 18-0102) with 100% ML and 1.00 PP bootstrap support (Fig. 1). The interspecies genetic distances between *A. mimusopis* (MFLU 25-0076) and *A. flava* (MFLU 18-0102) showed the following base pair differences (without gaps): 1.4% across LSU (13/892 bp) and 6.7% across ITS (37/530 bp). *Amphisphaeria mimusopis* can be distinguished from *A. flava* by having larger ascospores (18–22 × 5–7 µm vs 13–16 × 5–7μm) (Samarakoon et al. 2019). *Amphisphaeria mimusopis* has narrower paraphyses (3–6 µm) than *A. flava* (7–16.3 µm) (Samarakoon et al. 2019). The ascomata of *A. mimusopis* produce a pale reddish-brown pigment surrounding the ostioles, whereas *A. flava* results in a pale-yellow pigmentation on the surface (Samarakoon et al. 2019). Based on morphological and phylogenetic evidence, as well as the recommendations for species delineation by Chethana et al. (2021), we establish *Amphisphaeria mimusopis* as a new species.

#### ﻿ Amphisphaeria paraserianthis

Taxon classificationFungiAmphisphaerialesAmphisphaeriaceae

Z.L. Tun & K.D. Hyde sp. nov.

80565701-C43E-5DF1-AFAF-02E95634C9B0

Index Fungorum: IF903724

Facesoffungi Number: FoF17661

Fig. 5

##### Etymology.

The epithet refers to the host genus, *Paraserianthes*, from which the fungus was isolated.

**Figure 5.**
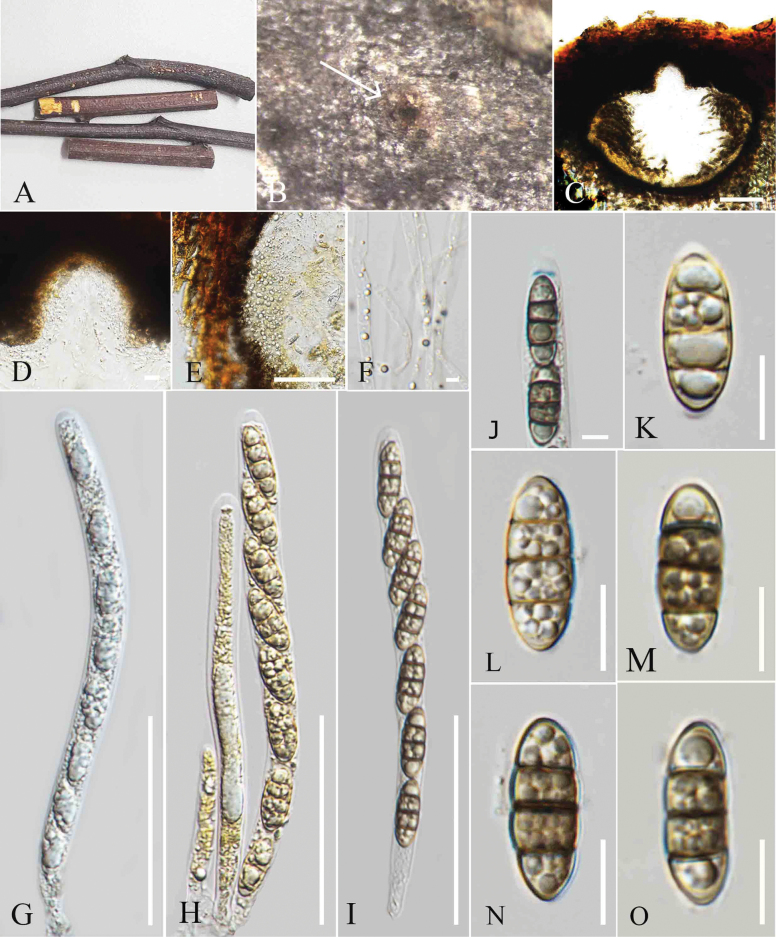
*Amphisphaeria paraserianthis* (MFLU 25-0075, holotype). A. Dead twigs of *Paraserianthes lophantha*; B. Appearance of ascomata on the host surface (arrow indicates the appearance of ascomata on the host surface); C. Vertical section of an ascoma; D. Ostiole; E. Peridium; F. Paraphyses; G–I. Asci; J. J+, apical ring turning blue in Melzer’s reagent; K–O. Immature and mature ascospores. Scale bars: 200 μm (C); 50 μm (D); 20 μm (E); 5 μm (F, J); 50 μm (G–I); 10 μm (J–O).

##### Holotype.

MFLU 25-0075.

##### Description.

***Saprobic*** on decaying twigs of *Paraserianthes lophantha*. **Sexual morph**: ***Ascomata*** 430–520 µm wide, 290–400 µm high (xˉ = 497 × 355 µm, n = 5), immersed, solitary or grouped, scattered, globose to subglobose, dark brown to black, ostiolate. ***Ostiole*** central, comprising a short papilla, with an ostiolar canal lined with hyaline *periphyses*. ***Peridium*** 16–20 µm (xˉ = 16 µm, n = 5) two-layered; outer layer wide, dark brown, thick-walled cells of ***textura angularis***, inner layer comprising one layer of pale yellow or sub-hyaline to hyaline cells of ***textura angularis***, thin-walled. ***Paraphyses*** 3–5 µm wide (xˉ = 4.5 µm, n = 5), hyaline, filiform, septate, guttulate, longer than asci. ***Asci*** 85–152 × 7.5–16 µm (xˉ = 125.5 × 10.5 µm, n = 20), 8-spored, unitunicate, cylindrical, thin-walled, short-pedicellate, apically rounded, with a J+, conspicuous, discoid, apical ring. ***Ascospores*** 16–20 × 5–9 µm (xˉ = 19 × 6.9 µm, n = 20), uniseriate, oblong to ellipsoid, hyaline when young, turning yellow brown at maturity, 3-septate, rounded to obtuse ends, smooth-walled, guttulate, without a sheath. **Asexual morph**: Not observed.

##### Material examined.

Thailand • Chiang Mai Province, in the forests around the Mushroom Research Center (19°07.200'N, 98°44.044'E), on fallen decaying twigs *of Paraserianthes lophantha* (Fabaceae), 14 November 2022, Zaw Lin Tun M14, (holotype MFLU 25-0075).

##### Notes.

*Amphisphaeria paraserianthis* (MFLU 25-0075) formed a distinct lineage that is basal to *A. neoaquatica* (MFLUCC 14-0045) and *A. hongheensis* (GMB1135) (Fig. 1). The interspecies genetic distances between *A. paraserianthis* and *A. neoaquatica* (MFLUCC 14-0045) showed the following base pair differences (without gaps): 7% for LSU (58/818 bp) and 5.11% for ITS (25/489 bp). *Amphisphaeria paraserianthis* and *A. hongheensis* (GMB1135) showed the following base pair differences (without gaps): 2.8% for LSU (26/923 bp) and 7.7% for ITS (35/455 bp). The ascomata of *A. paraserianthis* (430–520 × 290–400 µm) are larger than those of *A. neoaquatica* (250–320 × 300–330 μm) and smaller than those of *A. hongheensis* (430–750 × 360–640 μm). The asci of *A. paraserianthis* (85–152 × 7.5–16 µm) are larger than those of *A. neoaquatica* (126–138 µm × 8–10 μm), and smaller than *A. hongheensis* (175−265 × 10–15 µm) (Luo et al. 2019, Liu et al. 2024a). The ascospores of *A. paraserianthis* (16–20 × 5–9 µm) are larger than *A. neoaquatica* (15–17 × 5–7 μm) and smaller than *A. hongheensis* (20−40 × 5−11μm) µm (Luo et al. 2019, Liu et al. 2024a). In addition, *A. paraserianthis* possesses 3-septate ascospores while *A. neoaquatica* and *A. hongheensis* possess 1-septate ascospores (Luo et al. 2019, Liu et al. 2024a). Based on the species delineation suggestions made by Chethana et al. (2021) and the findings from morpho-phylogenetic analyses, we establish *A. paraserianthis* (MFLU 25-0075) as a new species.

#### ﻿ Amphisphaeria pseudomicheliae

Taxon classificationFungiAmphisphaerialesAmphisphaeriaceae

Z. L. Tun & K.D. Hyde sp. nov.

D78D68D1-A966-5F25-9819-7D7824AA83D1

Index Fungorum: IF903743

Facesoffungi Number: FoF17662

Fig. 6

##### Etymology.

Refers to the morphological similarity with *Amphisphaeria micheliae*.

**Figure 6.**
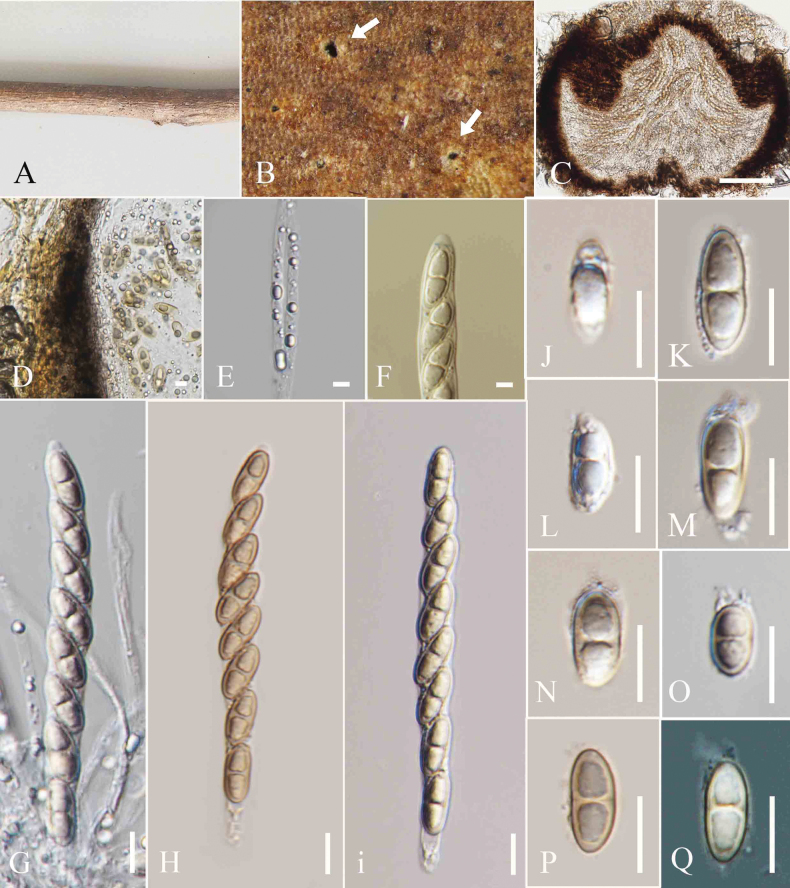
*Amphisphaeria pseudomicheliae* (MFLU 25-0077, holotype). A. Host; B. Appearance of ascomata on host (arrows indicate ascomata as tiny pores on host surface); C. Vertical section through an ascoma; D. Vertical section of peridium; E. Paraphyses; F. An inconspicuous J+ apical ring; G–I. Asci; J–Q. Ascospores; Q. An ascospore with a thin gelatinous sheath (in Indian ink). Scale bars: 100 μm (C); 10 μm (D–F); 20 μm (G–I); 10 μm (J–Q).

##### Holotype.

MFLU 25-0074.

##### Description.

***Saprobic*** on decaying twigs. **Sexual morph**: ***Ascomata*** 202–273 μm high, 349–400 µm diam (xˉ = 246 × 337 µm, n = 5), immersed, visible as tiny pores, flat, solitary to aggregated, scattered, globose to subglobose, brown, ostiolate. ***Peridium*** 17–23 µm wide (xˉ = 20.6 µm, n = 5), two-layered; outer layer wide, dark brown, comprising thick-walled cells of ***textura angularis***, inner layer comprising hyaline cells of ***textura angularis***, thin-walled. ***Paraphyses*** 4–4.4 µm (xˉ = 4.2 µm, n = 5) wide, hyaline, longer than asci, filiform, guttulate, septate, embedded in a gelatinous matrix. ***Asci*** 84–97× 7–9 µm (xˉ = 89.8 × 8.45 µm, n = 20), 8-spored, unitunicate, cylindrical, with short pedicel, apically rounded and narrowed, with a J+ inconspicuous apical ring. ***Ascospores*** 11–17 × 4–6 µm (xˉ = 15 × 5 µm, n = 20), uniseriate, fusiform, hyaline when immature, turning yellow to yellowish brown on maturity, 1-septate, guttulate, smooth-walled, slightly constricted at septum, straight to slightly curved, surrounded by a thin mucilaginous sheath. **Asexual morph**: Not observed.

##### Material examined.

Thailand • Chiang Rai Province, Mae Fah Luang University premises (20°02′42″N, 99°53′41″E), on decaying dead twigs of an undetermined host, 06 July 2023, Zaw Lin Tun E15 (holotype MFLU 25-0074).

##### Notes.

*Amphisphaeria pseudomicheliae* is closely related to *A. micheliae*, receiving robust support with 97% ML and 1 PP bootstrap support (Fig. 1). However, it can be distinguished from *A. micheliae* by its larger ascomata (202–273 × 349–400 µm vs. 180–210 × 225–370 µm) and smaller asci (84–97 × 7–9 µm vs. 92–135 × 7–10.5 μm) (Samarakoon et al. 2020). Additionally, the ascospores of *A. pseudomicheliae* are smaller (11–17 × 4–6 µm) than those of *A. micheliae* (15.5–21 × 6–7.5 μm) (Samarakoon et al. 2020). *Amphisphaeria pseudomicheliae* has a thin mucilaginous sheath, whereas *A. micheliae* lacks (Samarakoon et al. 2020). The genetic analysis reveals interspecies distances of 4.3% base pair differences (without gaps) in the ITS (23/524 bp) and 0.43% in the LSU (4/873 bp) between *A. pseudomicheliae* (MFLU25-0074) and *A. micheliae* (HKAS 107012). *Amphisphaeria micheliae* is uncertain since our new species lack protein coding genes. Thus, numerous collections with the protein coding genes can provide better resolution between the species. *Amphisphaeria pseudomicheliae* is described as a new species based on its distinct morphology and phylogenetic data.

#### ﻿ Amphisphaeria pterocarpi

Taxon classificationFungiAmphisphaerialesAmphisphaeriaceae

Z.L. Tun & K.D. Hyde sp. nov.

A235A1BC-E8B3-51B8-BC50-CF2EE7E4E8A9

Index Fungorum: IF903750

Facesoffungi Number: FoF17663

Fig. 7

##### Etymology.

The epithet refers to the host genus *Pterocarpus*, from which the fungus was isolated.

**Figure 7.**
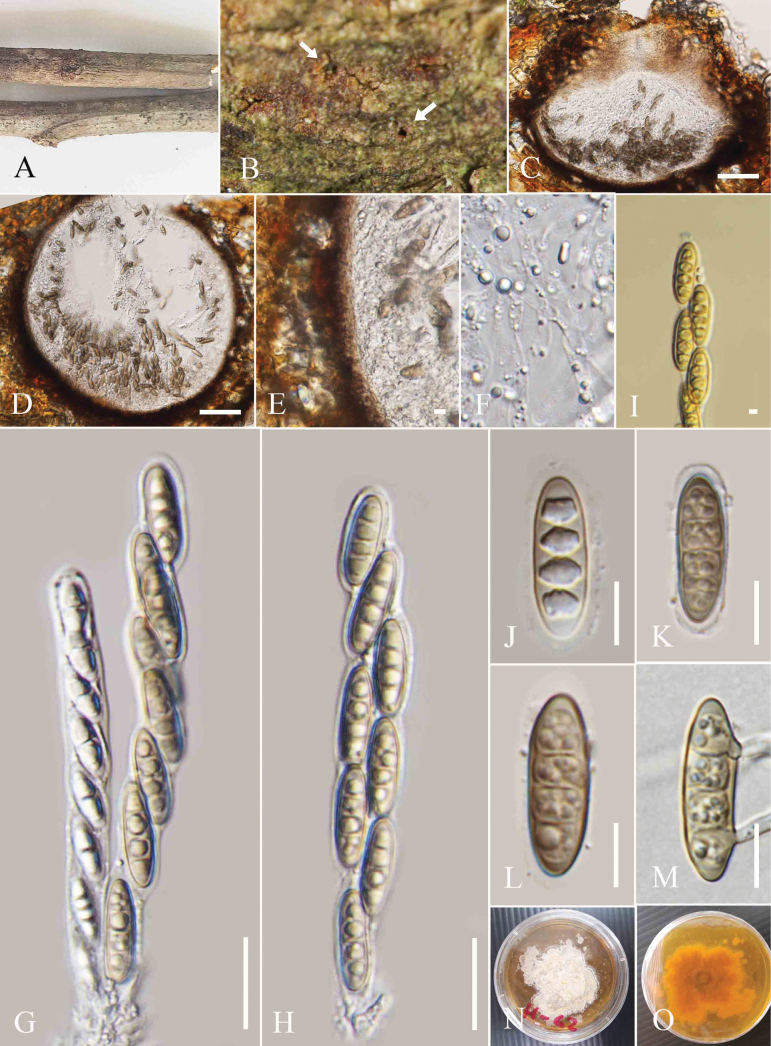
*Amphisphaeria pterocarpi* (MFLU 25-0073, holotype). A. Dead branches; B. Appearance of ascomata on host (arrows indicate ascomata as spots on host surface); C, D. Vertical sections through ascomata; E. Vertical section of peridium; F. Paraphyses; G, H. Asci; I. J+ apical ring; J–L. Ascospores; M. Germinated ascospore; N. Upper view of culture; O. Reverse view of culture. Scale bars: 100 μm (C, D); 10 μm (E); 5 μm (F, G); 50 μm (H, I); 20 μm (J–M).

##### Holotype.

MFLU 25-0073.

##### Description.

***Saprobic*** on recently dead branches of *Pterocarpus* sp. **Sexual morph. *Ascomata*** 225–237 μm high, 355–373 µm wide, (xˉ = 231 × 360 µm, n = 5), immersed, visible as black spots, host tissue becoming reddish around the apical pores, solitary to aggregated, scattered, globose to sub-globose, brown. ***Ostiole*** central, 70–60 wide, comprising a short papilla. ***Peridium*** 12–16 µm wide (xˉ = 14 µm, n = 5), two-layered; outer layer wide, dark brown, thick-walled cells of ***textura angularis***, inner layer comprising one layer of hyaline cells of ***textura angularis***, thin-walled. ***Paraphyses*** 4–6 µm wide (xˉ = 4.8 µm, n = 5), hyaline, septate, guttulate, embedded in a gelatinous matrix. ***Asci*** 95–116 × 9–14 µm (xˉ = 106 × 11 µm, n = 20), 8-spored, unitunicate, cylindrical, deliquescing, with short pedicel, apically rounded, with a J+, wedge-shaped, apical ring. ***Ascospores*** 19–21 × 5–7 µm (xˉ = 20 × 6 µm, n = 20), ellipsoidal, hyaline when immature, turning yellow to yellowish-brown when mature, 3-septate, guttulate, smooth-walled, surrounded by a mucilaginous sheath. **Asexual morph**: Not observed.

##### Culture characteristics.

Colonies on MEA reaching 4 cm diam. after 15 days at 27 °C, from above white, dense, irregular, flattened with smooth surface, with lobate margin; reverse yellow red in the middle, yellow at the margin.

##### Material examined.

Thailand • Chiang Rai Province, Mae Fah Luang University premises (20°02′42″N, 99°53′41″E), on recently dead branches of *Pterocarpus rotundifolius* (Fabaceae), 06 November 2023, Zaw Lin Tun H62 (holotype MFLU 25-0073), ex-type culture MFLUCC 25-0195.

##### Additional specimens examined.

Thailand • Chiang Rai Province, Mae Fah Luang University premises (20°02′42″N, 99°53′41″E), on dead branches of *Pterocarpus rotundifolius* (Fabaceae), 06 November 2023, Zaw Lin Tun 2H62 (MFLU 25-0072).

##### Notes.

Based on our phylogenetic analyses, *A. pterocarpi* formed a separate lineage, sister to *A. curvaticonidia* (MFLUCC 18-0620, HKAS 102288), with 100% ML and 1.00 PP bootstrap support (Fig. 1). *Amphisphaeria pterocarpi* can be distinguished from *A. curvaticonidia* by its smaller ascomata (225–237 × 355–373 µm vs. 320–390 × 360–410 µm), asci (95–116 × 9–14 µm vs. 121–162 × 10.5–17.5 µm), and ascospores (19–21 × 5–7 µm vs. 17–23 × 6–9 µm) (Samarakoon et al. 2020). Additionally, the ascospores of *A. pterocarpi* are ellipsoidal, whereas *A. curvaticonidia* have oblong or narrowly fusiform ascospores. The asexual morph of *A. curvaticonidia* has been documented as coelomycetous in culture, while the asexual morph of *A. pterocarpi* has yet to be observed in culture (Samarakoon et al. 2020). When considering the base pair differences (without gaps) between *A. pterocarpi* and *A. curvaticonidia* (MFLUCC 18-0620, HKAS 102288), 2.5% base pair differences (without gaps) were revealed in LSU (27/1040 bp) and 3.30% base pair differences (without gaps) in ITS (18/545 bp). Due to the distinct morphology and phylogenetic evidence, along with the species delineation guidelines provided by Chethana et al. (2021), we introduce *A. pterocarpi* as a new species.

#### ﻿ Amphisphaeria schimae

Taxon classificationFungiAmphisphaerialesAmphisphaeriaceae

Z.L. Tun & K.D. Hyde sp. nov.

18EC6EC8-7863-5BA8-ACB4-06C7E18BEE93

Index Fungorum: IF903771

Facesoffungi Number: FoF17664

Fig. 8

##### Etymology.

The epithet refers to the host genus, *Schima*, from which the fungus was isolated.

**Figure 8.**
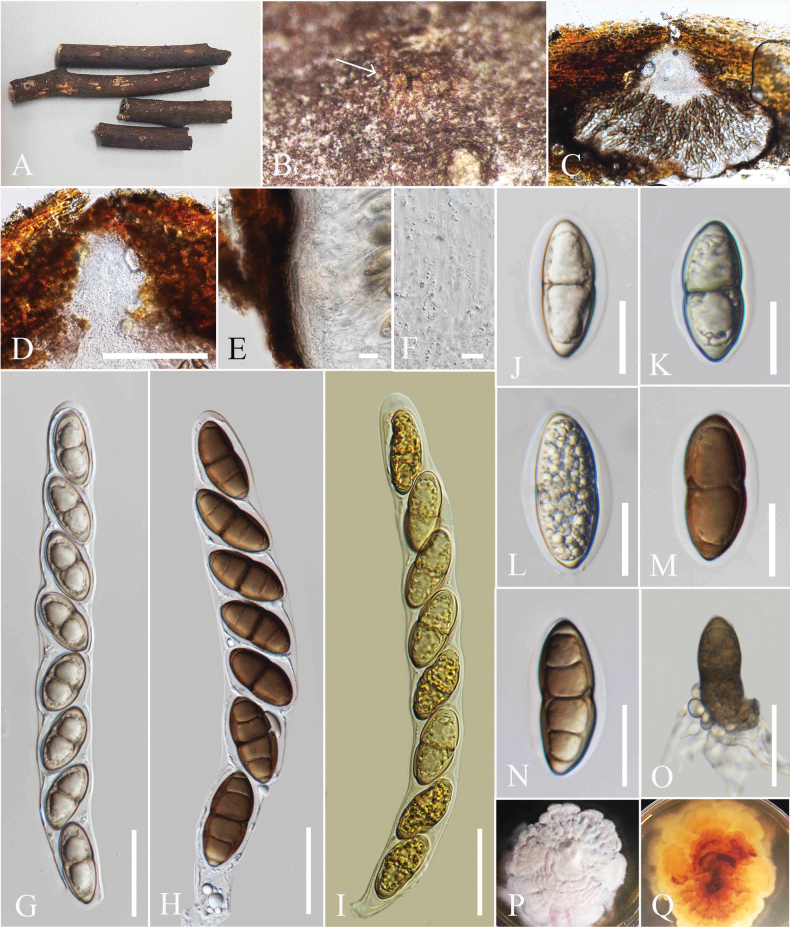
*Amphisphaeria schimae* (MFLU 25-0070, holotype). A. Host; B. Close-up of ascoma on host (Arrow indicates ascoma visible as a black spot, with ostiole breaking through the host); C. Vertical section of an ascoma; D. Ostiole; E. Vertical section of peridium; F. Paraphyses; G–I. Asci (**I** in Melzer’s reagent); J–N. Ascospores with sheaths; O. Germinated ascospore; P. Upper view of culture; Q. Reverse view of culture. Scale bars: 100 μm (C); 20 μm (D); 10 μm (E, F); 50 μm (G–I); 20 μm (J–O).

##### Holotype.

MFLU 25-0070.

##### Description.

***Saprobic*** on decaying twigs of *Schima wallichii*. **Sexual morph**: ***Ascomata*** 480–620 μm high × 520–683 µm wide, (xˉ = 531 × 583 µm, n = 5), immersed with ostiole breaking through host surface, visible as black spot, solitary to aggregated, scattered, globose to subglobose, brown, ostiolate. ***Ostiole*** central, comprising a short papilla, with an ostiolar canal. ***Peridium*** 13–16 µm wide (xˉ = 14.2 µm, n = 5), two-layered; outer layer thick, dense, reddish-brown cells of ***textura angularis***, inner layer thin, hyaline cells of ***textura angularis***. ***Paraphyses*** 3–5 µm wide, hyaline, filiform, septate, embedded in a gelatinous matrix. ***Asci*** 196–275 × 21–30 µm (xˉ = 236.2 × 22.9 µm, n = 20), 8-spored, unitunicate, cylindrical, with short pedicel, apically rounded, with J-, apical ring. ***Ascospores*** 36.9–40 × 13.5–15 µm (xˉ = 38.6 × 14 µm, n = 20), uniseriate, broadly fusiform, hyaline, turning olivaceous brown to brown at maturity, one median and constricted euseptum, with two distosepta, guttulate (especially at immaturity), broad to conically rounded at both ends, smooth, surrounded by a thick mucilaginous sheath. **Asexual morph**: Not observed.

##### Culture characteristics.

Colonies on MEA slow-growing, reaching 3 cm in diam. after 15 days at 27 °C, from above white to pale yellow radiating outwards, dense, circular, irregular, flattened with smooth surface, with lobate margin; reverse yellow brown in the middle, white at the margin.

##### Materials examined.

Thailand • Chiang Mai Province, in forests around the Mushroom Research Center (19°07.200'N, 98°44.044'E), on fallen dead twigs of *Schima wallichii* (Theaceae), 14 November 2022, Zaw Lin Tun C1 (holotype MFLU 25-0070), ex-type culture MFLUCC 25-0196.

##### Additional specimens examined.

Thailand • Chiang Mai Province, in forests around the Mushroom Research Center (19°07.200'N, 98°44.044'E), on fallen dead twigs of *Schima wallichii* (Theaceae), 14 November 2022, Zaw Lin Tun C2 (MFLU 25-0071).

##### Notes.

Based on our phylogenetic analyses, *Amphisphaeria schimae* (MFLU 25-0071, MFLUCC 25-0196) is sister to *A. ailaoshanensis* (KUNCC 23-15520, KUNCC 23-15521) (Fig. 1). However, *A. schimae* can be distinguished from *A. ailaoshanensis* by having larger ascomata (480–620 μm high × 520–683 μm vs. 100–140 μm high × 250–350 μm), asci (196–275 × 21–30 μm vs. 70–100 × 7–10 μm), and ascospores (36.9–40 × 13.5–15 μm vs. 14–20 × 5–8 μm) (Dissanayake et al. 2024). Additionally, the ascospores of *A. schimae* are broadly fusiform, hyaline, turning olivaceous brown to brown at maturity, with one median, conspicuously constricted euseptum, and surrounded by a thick mucilaginous sheath, whereas those of *A. ailaoshanensis* are fusiform, hyaline, guttulate, turning brown, 1–3-septate, and lack a mucilaginous sheath (Dissanayake et al. 2024). When comparing base pair differences (excluding gaps) between *A. schimae* (MFLUCC 25-0196) and *A. ailaoshanensis* (KUNCC 23-15520, KUNCC 23-15521), there is a 4.9% divergence in the LSU region (34/690 bp) and 8.2% divergence in the ITS region (43/525 bp). Based on the distinct morphological and phylogenetic evidence, along with the species delineation guidelines proposed by Chethana et al. (2021), we describe *A. schimae* as a new species.

## ﻿Discussion

In this study, we introduce six new *Amphisphaeria* species, along with a new host and geographical record, based on morphological and multigene phylogenetic analyses of combined LSU and ITS alignments. This work significantly advances the taxonomy of *Amphisphaeria* by providing important insights into species delimitation, phylogenetic relationships, and morphological diversity. Moreover, the discovery of a new host and geographic record broadens our understanding of the ecological distribution and host specificity of these species, offering a more comprehensive framework for future taxonomic and ecological research in this group.

Most strains of *Amphisphaeria* lack protein gene sequences, with the exception of *A. camelliae*, *A. flava*, *A. fuckelii*, *A. hongheensis*, *A. hydei*, *A. micheliae*, *A. parvispora*, *A. sambuci*, *A. thailandica*, and *A. uniseptata* (Tusi et al. 2001; Jaklitsch et al. 2016; Voglmayr et al. 2019; Liu et al. 2019, 2024a; Samarakoon et al. 2019; Samarakoon 2023; Li et al. 2024). In this study, we encountered challenges in sequencing protein markers across our *Amphisphaeria* isolates. Specifically, all isolates lacked the *β-tub* and the *rpb*2 gene. These difficulties align with findings from previous studies, which also reported challenges in sequencing these protein markers for *Amphisphaeria* (Senanayake et al. 2019; Samarakoon et al. 2020; Zhang et al. 2023; Dissanayake et al. 2024; Sun et al. 2025).

To address these limitations, we constructed two phylogenetic trees: one using a combination of LSU, ITS, *rpb*2, and *β-tub* (not shown), and another using only LSU and ITS (Fig. 1). Comparison of the topologies of the two-locus (LSU and ITS) and four-locus trees showed mostly similar phylogenetic placements of the taxa. Since most of our species lacked *β-tub* and *rpb2*, we predominantly relied on LSU and ITS for our phylogenetic analyses. This approach allowed us to achieve robust phylogenetic resolution despite the absence of key protein markers in most isolates.

Currently, 312 names are listed under *Amphisphaeria* in Index Fungorum (July 2025). Wang et al. (2004) examined 170 type specimens and accepted only 12 species in *Amphisphaeria*, highlighting taxonomic confusion within this genus. The lack of molecular data for many *Amphisphaeria* species makes it difficult to confirm their taxonomic positions (Thiyagaraja et al. 2025). Additionally, the absence of protein gene sequences for *Amphisphaeria* species often leads to inaccurate identifications (Samarakoon 2023; Dissanayake et al. 2024). The high morphological similarity and lack of molecular data among *Amphisphaeria* species can cause misidentifications (Thiyagaraja et al. 2025). Therefore, incorporating new collections and multi-gene molecular data is essential for accurate species delimitation and a clearer understanding of species boundaries within *Amphisphaeria*.

The saprobes were collected from forest areas during both the wet and cold seasons of 2022–2024. The distribution of *Amphisphaeria* species is shown in Table 3. *Amphisphaeria* species are found worldwide, with reports from 15 countries. They occur on 24 host genera across 17 different plant families, with Sapindaceae being the most diverse (Table 3). Additionally, *Amphisphaeria* has been reported in other families, including two species each in Theaceae, Leguminosae, and Malvaceae, three in Fabaceae, and one each in Proteaceae, Gramineae, Calophyllaceae, Magnoliaceae, Actinidiaceae, Apocynaceae, Rutaceae, Oleaceae, Asparagaceae, Agavaceae, Rosaceae, and Euphorbiaceae (Table 3). Therefore, Malvaceae and Fabaceae are the second most diverse families for *Amphisphaeria* species (Table 3).

**Table 3.** Accepted species in *Amphisphaeria*, including their host and geographical location (NA = Lack of information).

Species	Host	Country	References
*Amphisphaeria acericola*	On a branch of *Acer campestre* L. (Sapindaceae)	Italy	Samarakoon et al. (2019)
*A. ailaoshanensis*	NA	China	Dissanayake et al. (2024)
*A. aquatica*	NA	China	Luo et al. (2019)
*A. bertiana*	In cavities at the end of a rotting log	USA	Wang et al. (2004)
*A. camelliae*	*Camellia japonica* (Theaceae)	China	Samarakoon et al. (2020)
*A. chiangmaiensis*	NA	Thailand	Samarakoon (2023)
*A. curvaticonidia*	NA	Thailand	Samarakoon et al. (2020)
*A. depressa*	*Cassia bicapsularis* (Leguminosae)	USA,	Petrak 1953; Wang et al. (2004)
*A. falcata*	The medullary tissue of the lichen *Usneadiffracta*	China	Chang et al. (2025)
*A. fallax*	*Quercus robur* (Leguminosae)	Austria, Germany	De not 1865; Wang et al. (2004)
*A. flava*	NA	Thailand	Samarakoon et al. (2019)
*A. fuckelii*	On attached branches of *Tilia cordata*, *Acer campestre* (Malvaceae, Sapindaceae)	Germany, Austria, Belgium	Jaklitsch et al. (2016); Liu et al. (2019); Voglmayr et al. (2019)
*A. gaubae*	Dead leaves of *Lambertia formosa* (Proteaceae)	Australia	Wang et al. (2004)
*A. guttulata*	NA	Thailand	Liu et al. (2024b)
*A. hibiscicola*	*Hibiscus mutabilis* (Malvaceae)	China	Sun et al. (2025)
*A. hongheensis*	NA	China	Liu et al. (2024a)
*A. hydei*	NA	Thailand	Samarakoon (2023)
*A. karsti*	NA	China	Zhang et al. (2023)
*A. kunmingensis*	NA	China	Dissanayake et al. (2024)
*A. lusitanica*	*Arundo donax* (Gramineae)	Portugal	Wang et al. (2004)
*A. magna*	NA	China	Dissanayake et al. (2024)
*A. mesuae*	*Mesua* sp. (Calophyllaceae)	Thailand	This study
*A. micheliae*	*Alstonia scholaris*, *Acer truncatum*, on a dead branch of *Michelia alba*, *Senna siamea*, *Micromelum integerrimum* (Apocynaceae, Fabaceae, Magnoliaceae, Sapindaceae, Rutaceae)	China, Thailand	Samarakoon et al. (2020); Li et al. (2024); Pathirana et al. (2025), This study
*A. mimusopis*	*Mimusops elengi* (Sapotaceae)	Thailand	This study
*A. multipunctate*	*Ctinidia deliciosa* (Actinidiaceae)	New Zealand	Petrak (1923); Wang et al. (2004)
*A. oleae*	On branches of *Olea europaea* (Oleaceae)	China	Li et al. (2024)
*A. orixae*	The healthy roots of *Orixa japonica* (Asparagaceae)	China	Wang et al. (2023)
*A. paedida*	NA	Germany	Saccardo (1882); Wang et al. (2004)
*A. paraserianthis*	*Paraserianthes lophantha* (Fabaceae)	Thailand	This study
*A. parvispora*	NA	China	Samarakoon et al. (2022)
*A. pseudomicheliae*	NA	Thailand	This study
*A. pseudoumbrina*	On bark of *Acer campestre* (Sapindaceae)	Italy	Wang et al. (2004)
*A. pterocarpi*	*Pterocarpus rotundifolius* (Fabaceae)	Thailand	This study
*A. qujingensis*	NA	China	Dissanayake et al. (2020)
*A. sambuci*	*Sambucus nigra* (Adoxaceae)	England, France, Germany	Liu et al. (2019); Jaklitsch et al. (2016)
*A. schimae*	*Schima wallichii* (Theaceae)	Thailand	This study
*A. seriata*	On leaf of *Nolina* sp. (Agavaceae)	USA	Barr and Ramaley (1996); Wang et al. (2004)
*A. shangrilaensis*	NA	China	Dissanayake et al. (2024)
*A. sorbi*	On branch of *Sorbus aucuparia* L. (Rosaceae)	Italy	Liu et al. (2015)
*A. thailandica*	NA	Thailand	Samarakoon et al. (2019)
*A. umbrina*	*Tilia* sp. (Malvaceae)	Switzerland	Jeewon and Hyde (2003)
*A. uniseptata*	NA	China	Tusi et al. (2001)
*A. verniciae*	Branches of *Vernicia fordii* (Euphorbiaceae)	China	Li et al. (2024)
*A. vibratilis*	On the stem of *Prunus* sp. (Rosaceae)	Canada, British, Columbia	Müller and Arx (1962); Wang et al. (2004)
*A. xishuangbannaense*	NA	China	Dissanayake et al. (2024)
*A. yunnanensis*	NA	China	Dissanayake et al. (2020)

*Amphisphaeria chiangmaiensis*, *A. curvaticonidia*, *A. flava*, *A. hydei*, *A. parvispora*, *A. micheliae*, and *A. thailandica* have been previously documented in northern Thailand (Samarakoon et al. 2019–2023; Pathirana et al. 2025), highlighting the region as a hotspot for fungal diversity. In our study, we further contribute to the understanding of *Amphisphaeria* species in this region by describing six new species, each associated with different host families, as shown in Table 3. This discovery not only broadens the known taxonomic range of *Amphisphaeria* but also underscores the ecological adaptability of this genus to various plant hosts. Additionally, we report *A. micheliae* on *Senna siamea* for the first time in Thailand, marking an important addition to the fungal flora of the region. These findings collectively highlight the impressive diversity of *Amphisphaeria* species in northern Thailand and their associations with a wide array of host families, indicating a complex and dynamic ecological interaction that warrants further study. This genus also needs to be studied in other tropical regions to determine if it is equally diverse there (Hyde et al. 2024b).

Historically, there have been no records of *Amphisphaeria* species being identified as pathogens (Wang et al. 2004). However, recent studies have revealed new insights into the ecological roles of these fungi (Sun et al. 2025). For instance, *A. hibiscicola* was reported on diseased leaves of *Hibiscus mutabilis* (Sun et al. 2025). Additionally, *A. orixae* has been identified as an endophyte, highlighting its ability to colonize plant tissues without causing disease (Wang et al. 2023). These findings indicate that *Amphisphaeria* species may exhibit diverse ecological roles, ranging from pathogenicity to endophytism, and may have evolved from endophytic species (Bhunjun et al. 2024). Furthermore, while most *Amphisphaeria* species are typically found in terrestrial environments (Dissanayake et al. 2024), the discovery of *Amphisphaeria aquatica* in freshwater habitats on submerged, decaying wood in China and Thailand (Tsui et al. 2001; Luo et al. 2019) expands our understanding of their ecological adaptability. This contrast between terrestrial and aquatic habitats highlights the ecological versatility of the genus and raises questions about how environmental factors influence their behavior and role in ecosystems. Therefore, future research should concentrate on clarifying the complex ecological interactions between these fungi and their hosts, as well as examining the impact of environmental factors on their distribution and behavior. Furthermore, it is essential to enhance the understanding of the ecological and evolutionary importance of *Amphisphaeria* species. This acknowledges the ongoing significance of northern Thailand as a vital center for continued research, as emphasized by Hyde et al. (2018).

## Supplementary Material

XML Treatment for Amphisphaeria mesuae

XML Treatment for Amphisphaeria micheliae

XML Treatment for Amphisphaeria mimusopis

XML Treatment for Amphisphaeria paraserianthis

XML Treatment for Amphisphaeria pseudomicheliae

XML Treatment for Amphisphaeria pterocarpi

XML Treatment for Amphisphaeria schimae

## ﻿Additional information

### Use of AI

No use of AI was reported.

### Author ORCIDs

Zaw Lin Tun https://orcid.org/0009-0001-3108-4425

Digvijayini Bundhun https://orcid.org/0000-0002-0790-215X

Chitrabhanu S. Bhunjun https://orcid.org/0000-0001-8098-3390

Sajeewa S. N. Maharachchikumbura https://orcid.org/0000-0001-9127-0783

Vinodhini Thiyagaraja https://orcid.org/0000-0002-8091-4579

Fatimah Al-Otibi https://orcid.org/0000-0003-3629-5755

Kevin D. Hyde https://orcid.org/0000-0002-2191-0762

### Data availability

The DNA sequences produced in this study are available on NCBI GenBank (https://www.ncbi.nlm.nih.gov). The vouchers are deposited in the the Mae Fah Luang University Herbarium and culture collection (MFLU, MFLUCC).

## Supplementary materials

Supplementary material 1 Supplementary data Data type zip https://binary.pensoft.net/file/1465224This dataset is made available under the Open Database License (http://opendatacommons.org/licenses/odbl/1.0/). The Open Database License (ODbL) is a license agreement intended to allow users to freely share, modify, and use this Dataset while maintaining this same freedom for others, provided that the original source and author(s) are credited.Zaw Lin Tun, Digvijayini Bundhun, Chitrabhanu S. Bhunjun, Sajeewa S. N. Maharachchikumbura, Vinodhini Thiyagaraja, Fatimah Al-Otibi, Kevin D. Hyde
