# Supplementary material for: ﻿The addition of six novel species and a new record of Amphisphaeria from northern Thailand
Source: MycoKeys. 2025 Nov 14;125:1–31. doi: 10.3897/mycokeys.125.163523 (PMC12639359; doi:10.3897/mycokeys.125.163523)
Supplement: Supplementary material 1 — Supplementary data [file mycokeys-125-001-s001.zip › LSU AND ITS CB supplementary file/4 gene tree.pptx]

## Slide 1
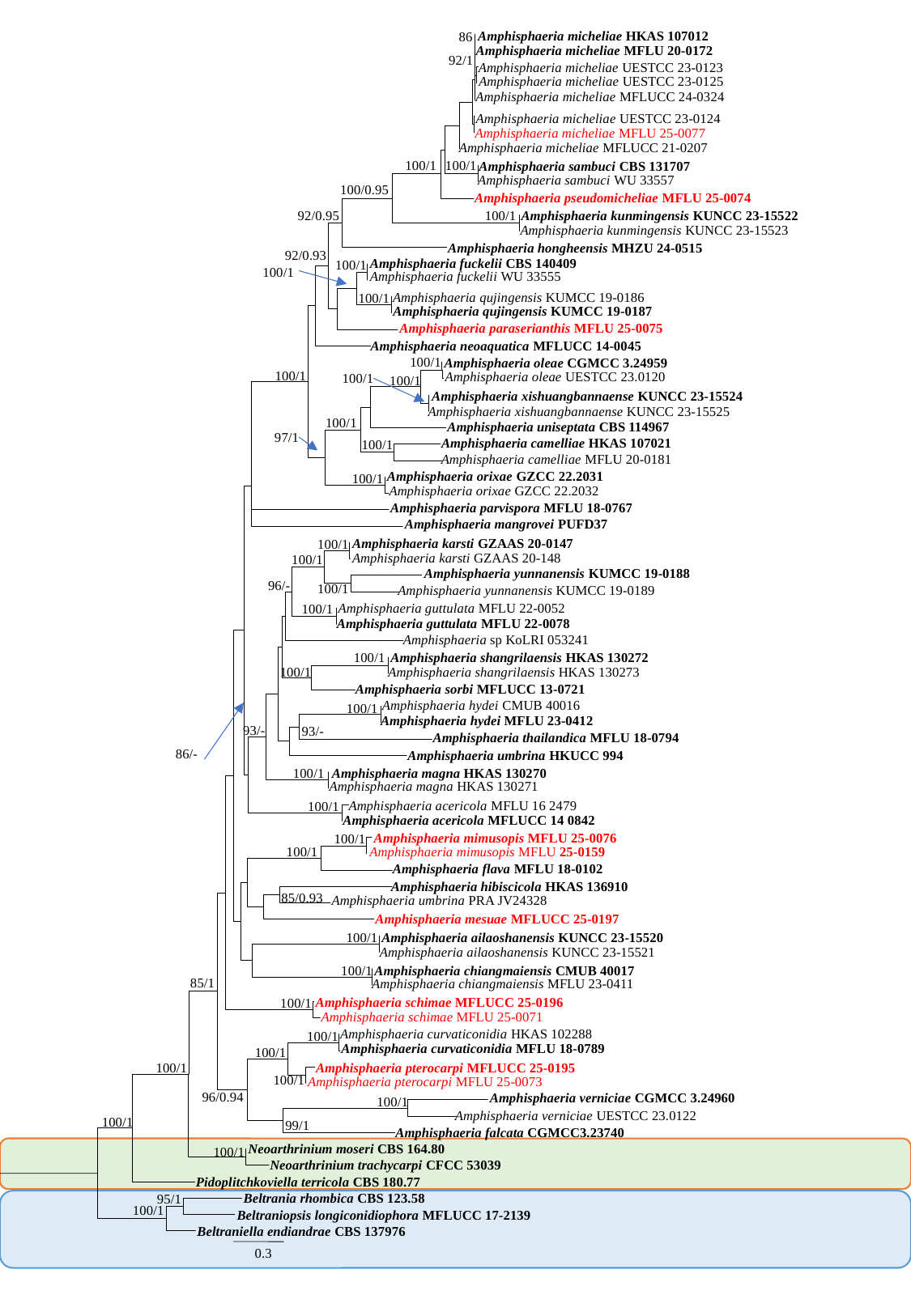

Amphisphaeria micheliae HKAS 107012
86
0.3
Amphisphaeria micheliae MFLU 20-0172
92/1
Amphisphaeria micheliae UESTCC 23-0123
Amphisphaeria micheliae UESTCC 23-0125
Amphisphaeria micheliae MFLUCC 24-0324
Amphisphaeria micheliae UESTCC 23-0124
Amphisphaeria micheliae MFLU 25-0077
Amphisphaeria micheliae MFLUCC 21-0207
100/1
100/1
Amphisphaeria sambuci CBS 131707
Amphisphaeria sambuci WU 33557
100/0.95
Amphisphaeria pseudomicheliae MFLU 25-0074
92/0.95
Amphisphaeria kunmingensis KUNCC 23-15522
100/1
Amphisphaeria kunmingensis KUNCC 23-15523
Amphisphaeria hongheensis MHZU 24-0515
92/0.93
Amphisphaeria fuckelii CBS 140409
100/1
100/1
Amphisphaeria fuckelii WU 33555
Amphisphaeria qujingensis KUMCC 19-0186
100/1
Amphisphaeria qujingensis KUMCC 19-0187
Amphisphaeria paraserianthis MFLU 25-0075
Amphisphaeria neoaquatica MFLUCC 14-0045
100/1
Amphisphaeria oleae CGMCC 3.24959
100/1
Amphisphaeria oleae UESTCC 23.0120
100/1
100/1
Amphisphaeria xishuangbannaense KUNCC 23-15524
Amphisphaeria xishuangbannaense KUNCC 23-15525
100/1
Amphisphaeria uniseptata CBS 114967
97/1
Amphisphaeria camelliae HKAS 107021
100/1
Amphisphaeria camelliae MFLU 20-0181
Amphisphaeria orixae GZCC 22.2031
100/1
Amphisphaeria orixae GZCC 22.2032
Amphisphaeria parvispora MFLU 18-0767
Amphisphaeria mangrovei PUFD37
Amphisphaeria karsti GZAAS 20-0147
100/1
Amphisphaeria karsti GZAAS 20-148
100/1
Amphisphaeria yunnanensis KUMCC 19-0188
96/-
100/1
Amphisphaeria yunnanensis KUMCC 19-0189
Amphisphaeria guttulata MFLU 22-0052
100/1
Amphisphaeria guttulata MFLU 22-0078
Amphisphaeria sp KoLRI 053241
100/1
Amphisphaeria shangrilaensis HKAS 130272
100/1
Amphisphaeria shangrilaensis HKAS 130273
Amphisphaeria sorbi MFLUCC 13-0721
Amphisphaeria hydei CMUB 40016
100/1
Amphisphaeria hydei MFLU 23-0412
93/-
93/-
Amphisphaeria thailandica MFLU 18-0794
86/-
Amphisphaeria umbrina HKUCC 994
100/1
Amphisphaeria magna HKAS 130270
Amphisphaeria magna HKAS 130271
Amphisphaeria acericola MFLU 16 2479
100/1
Amphisphaeria acericola MFLUCC 14 0842
Amphisphaeria mimusopis MFLU 25-0076
100/1
100/1
Amphisphaeria mimusopis MFLU 25-0159
Amphisphaeria flava MFLU 18-0102
Amphisphaeria hibiscicola HKAS 136910
85/0.93
Amphisphaeria umbrina PRA JV24328
Amphisphaeria mesuae MFLUCC 25-0197
100/1
Amphisphaeria ailaoshanensis KUNCC 23-15520
Amphisphaeria ailaoshanensis KUNCC 23-15521
100/1
Amphisphaeria chiangmaiensis CMUB 40017
85/1
Amphisphaeria chiangmaiensis MFLU 23-0411
Amphisphaeria schimae MFLUCC 25-0196
100/1
Amphisphaeria schimae MFLU 25-0071
Amphisphaeria curvaticonidia HKAS 102288
100/1
Amphisphaeria curvaticonidia MFLU 18-0789
100/1
Amphisphaeria pterocarpi MFLUCC 25-0195
100/1
100/1
Amphisphaeria pterocarpi MFLU 25-0073
96/0.94
Amphisphaeria verniciae CGMCC 3.24960
100/1
Amphisphaeria verniciae UESTCC 23.0122
100/1
99/1
Amphisphaeria falcata CGMCC3.23740
Neoarthrinium moseri CBS 164.80
100/1
Neoarthrinium trachycarpi CFCC 53039
Pidoplitchkoviella terricola CBS 180.77
Beltrania rhombica CBS 123.58
95/1
100/1
Beltraniopsis longiconidiophora MFLUCC 17-2139
Beltraniella endiandrae CBS 137976
